# Daytime contacts and general practitioner consultations, and pain as a reason for encounter in children with cerebral palsy; a Norwegian national registry linkage study

**DOI:** 10.1080/02813432.2022.2144992

**Published:** 2022-12-01

**Authors:** Selma Mujezinović Larsen, Torunn Bjerve Eide, Cathrine Brunborg, Kjersti Ramstad

**Affiliations:** a Faculty of Medicine, Institute of Clinical Medicine, University of Oslo and Division of Paediatric and Adolescent Medicine, Rikshospitalet, Oslo University Hospital, Oslo, Norway; b Department of General Practice, Institute of Health and Society, University of Oslo, Oslo, Norway; c Oslo Center for Statistics and Epidemiology, Oslo University Hospital, Oslo, Norway; d Division of Paediatric and Adolescent Medicine, Rikshospitalet, Oslo University Hospital, Oslo, Norway

**Keywords:** Cerebral palsy, pain, children, primary health care, reason for encounter, linkage study

## Abstract

**Aim:**

The aim of this study was to compare the prevalence of daytime contacts and consultations, and pain as a reason for encounter (RFE) with a general practitioner (GP), in children with cerebral palsy (CP) (cases) to that of the general paediatric population (controls).

**Methods:**

The study linked the Norwegian Directorate of Health’s database for the control and reimbursement of health expenses, and the Norwegian Quality and Surveillance Registry for Cerebral Palsy, including children born from 1996 to 2012 in the period 2006 to 2018. All daytime contacts were included. International Classification for Primary Care was applied for RFE.

**Results:**

Cases accounted for 0.46% of all daytime contacts and 0.27% of all daytime consultations, the latter corresponding with the estimated national prevalence of CP. GPs registered more administrative contact and coded pain as an RFE less frequently in consultations with cases (6%) than with controls (12%).

**Interpretation:**

Children with CP did not consult GPs more than the general paediatric population did. In consultations, GPs should ask for pain even if the child with CP or parent does not address pain. The local multidisciplinary team should encourage the family to consider consulting a GP if the child is in pain.

**KEY MESSAGES**
- Prevalence of GP consultations in children with CP is similar to that of children in the general population.
- GPs perform more administrative work for children with CP than for their other paediatric patients.
- GPs code pain as an RFE less frequently in consultations with children with CP than in consultations with children in the general population.

## Introduction

An increasing number of children live with chronic health conditions [1] requiring measures from a variety of health care providers [2]. In Norway, the health authorities affiliate every citizen with a general practitioner (GP) whom one can consult for current medical needs and who interacts both with other locally based professionals and with specialist care when necessary. The GPs’ position is unique and makes the GP a cornerstone in the network of care recommended for the management of chronic medical conditions. This continuity of primary care is associated with both lower morbidity and mortality in the general population [3]. Children under the age of 16 years do not pay a consultation fee, while for patients above 16 years an upper limit for personal annual health care costs is set [4]. This ensures affordable medical services for all inhabitants. Still, knowledge on GPs’ involvement in the management of chronic health conditions is scarce. Pain, both acute and chronic, is a health complaint managed often by Norwegian GPs [5].

Cerebral palsy (CP) is the most common chronic motor disorder in children [6], often accompanied by disturbances in sensation, perception, cognition, communication and behaviour, epilepsy and secondary musculoskeletal problems [7]. The prevalence of CP in Norway is 2.5 (95% CI 2.4 − 2.7) per 1000 [8]. The great variety of impairments and medical needs in CP, together with the emerging insights in disease trajectories from CP surveillance programs, makes CP a relevant model health condition in exploring GPs’ involvement in the care for children with chronic health conditions.

Pain is more common in children with CP than in the general paediatric population as about three of four children with CP report pain when asked [9,10], in contrast to about one in five to one in six in the general paediatric population [11,12]. While headache and abdominal pain top the pain sites list in the general paediatric population [12], musculoskeletal pain is dominating in the population with CP [13–15]. Causes of musculoskeletal pain in CP are muscle overuse, immobilization, strain caused by involuntary movements, atypical compression from the imbalance of muscle activation across joints and their combinations [16]. Further, abdominal pain has a high prevalence in children with severe CP [14]. The current opinion is that the high prevalence of pain in CP reflects health care deficiencies [17,18], and that studies on pain management are needed [19] to inform initiatives which aim to decrease pain.

We found no studies comparing contact with a GP between children with CP to that of the children from the general population. Thus, we chose to follow the general recommendation of the ‘neutral’ hypotheses even though we were aware that chronic disability might influence contact with a GP. In the present study, we compared the prevalence of daytime contacts and daytime consultations with a GP, and pain as a reason for encounter (RFE) in children with CP to that of the general paediatric population. The null hypotheses were:

1. The prevalence of daytime paediatric consultations for all reasons is not affected by CP diagnosis
2. The prevalence of daytime paediatric consultations because of pain is not affected by CP diagnosis
3. The prevalences of daytime paediatric consultations because of headache, abdominal, and musculoskeletal pain, are not affected by CP diagnosis.

## Methods

### Study design

The study compares a registry-based cohort of children with CP (cases) to the general population of the same age (controls), linking two national databases: KUHR, the Norwegian Directorate of Health's database for the control and reimbursement of health expenses [20], and the Norwegian Quality and Surveillance Registry for Cerebral Palsy (NorCP) [21].

### Data sources

World Health Organization (WHO) accepts International Classification for Primary Care (ICPC-2) as an RFE classification in primary care or general practice wherever applicable [22]. The World Organization of Family Doctors (WONCA) owns ICPC-2, and its use is license-based. ICPC-2 has been in use since 1998 in Norway, and in electronic format since 2002 [23]. The last revision of ICPC-2 was in 2003, and the KUHR registry holder assumes the correct use of the electronic version as of 2006. ICPC-2 consists of 17 chapters on organ systems. Each chapter includes codes for symptoms and complaints (numbers 01–29), process codes (numbers 30–69) and disease codes (numbers 70–99).

In Norway, GPs have an agreement on reimbursement of health expenses from the government through the KUHR database [20]. Reimbursement requires registration of the type of contact and at least one ICPC-2 code. The age at each contact is registered automatically due to the use of the national personal identification number.

Since 2004, all children with CP in Norway, born 1996 and later, are invited to register in NorCP and follow a CP surveillance program. CP diagnosis is confirmed by a paediatrician at the age of five years according to the algorithm given by the Surveillance of Cerebral Palsy in Europe [24]. Children with CP were identified in KUHR using the national personal identification number applied in both databases. To ensure a confirmed CP diagnosis, children born later than 2012 were not included in the study.

### Study population

All children born 1996–2012 registered in KUHR in the period 2006–2018 were included. Children registered in NorCP were cases, and children not registered in NorCP were controls.

### Variables

All contacts during afternoon and night, holidays and weekends were excluded to focus on GPs’ daytime work only. One reason for this choice is difference in organization of out-of-hour services in rural and urban areas. Outcome variables were number and type of GP daytime contacts, and number of ICPC-2 codes considered to reflect pain as an RFE.

Contacts during daytime were grouped using mutually exclusive reimbursement codes. Physical or electronic encounters with a GP (the latter in use from 2013), were labelled consultations, while activities not requiring direct contact between the GP and the patient were labelled administrative contacts. The latter include simple contacts (patient’s attendance at the medical center not meeting the GP), GPs’ interdisciplinary interactions with other professionals in primary care such as a meeting or a telephone call, referrals without consultation, and prescription renewals (in use from 2011 as a separate reimbursement code).

Analysis on pain as an RFE was performed in the consultations only. ICPC-2 codes regarded relevant for pain are listed in Table 1, and are in further text labelled ‘pain codes’. Codes from the ICPC-2 chapter ‘L Musculoskeletal’ are labelled ‘musculoskeletal pain’, and codes from other chapters are collapsed and labelled ‘other pain’. We also grouped the pain codes according to three most frequent anatomical pain sites: headache, abdominal and musculoskeletal pain. In cases, the ICPC-2 disease codes for CP (Neurological disorder N99) was included.

**Table 1.** Selected ICPC-2 codes from the chapters A, D, L, N, S and U grouped into ‘musculoskeletal pain’ and ‘other pain’.

‘Musculoskeletal pain’	‘Other pain’
L Musculoskeletal	A General
L01 Symptom/complaint in neck	A01 General pain
L02 Symptom/complaint in back	
L03 Symptom/complaint in low back	D Digestive
L04 Symptom/complaint in chest	D01 Abdominal pain
L05 Symptom/complaint in flank/axilla	D02 Abdominal pain epigastric
L07 Symptom/complaint in jaw	D04 Rectal/anal pain
L08 Symptom/complaint in shoulder	D06 Abdominal pain location other
L09 Symptom/complaint in arm	
L10 Symptom/complaint in elbow	N Neurological
L11 Symptom/complaint in wrist	N01 Headache
L12 Symptom/complaint in hand/finger	N95 Tension headache
L13 Symptom/complaint in hip	
L14 Symptom/complaint in leg/thigh	S Skin
L15 Symptom/complaint in knee	S01 Pain/tenderness skin
L16 Symptom/complaint in ankle	S29 Skin symptom/complaint
L17 Symptom/complaint in foot/toe	S97 Chronic ulcer skin
L18 Muscle pain	
L19 Symptom/complaint in muscle	U Urological
L20 Symptom/complaint in joint	U01 Painful urination
L29 Symptom/complaint in musculoskeletal other	U13 Bladder symptom/complaint
	U29 Urinary symptom/complaint

### Statistics

STATA version 16 (Stata Corp LLC, Texas, USA) was used for the statistical analysis. Data are presented as number and percentage of contacts. STATA calculator for cohort studies was used to calculate a risk ratio with 95% confidence interval (CI) for three age groups (0–5, 6–11 and 12–17 years), thus adjusting for age. Risk ratio below one means that cases had lower risk than controls, and above one that cases had higher risk than controls.

### Ethics

This study was approved by the Regional Committee for Medical and Health Research Ethics, South-East Norway (reference 2018/1250). National standards for storage and handling of data were applied to ensure privacy and protection.

### Registration

The study had been registered in Open Science Framework on 18 December 2019.

## Results

During the period 2006–2018, there were 23 616 791 daytime contacts, 108 413 (0.46%) in cases, and 23 508 378 (99.54%) in controls. The cases accounted for 0.27% of all 16 057 216 consultations. Cases accounted for 0.28, 0.30 and 0.21% of consultations in age groups 0–5, 6–11 and 12–17 years, respectively. Among the administrative contacts, cases accounted for 0.49% of all 4 785 643 simple contacts, 2.87% of all 469 953 interdisciplinary interactions, 1.37% of all 969 913 referrals, and 1.12% of all 1 334 066 prescriptions. The risks for a daytime contact being a simple contact, a GP’s interaction with other professionals, a referral, or a prescription, were higher in cases than in controls in all three age groups, except for simple contacts in the age group 12–17 years in which the risk was lower in cases than in controls (Table 2).

**Table 2.** Distribution of 23 616 791 daytime contacts in children with CP (cases) and children in general population (controls) according to the type of contact.

	Cases	Controls	Risk ratio (95% CI)
**Daytime contacts, age 0–5 years**	**35 855**	**8 855 216**	
Consultations	19 054 (53.1)	6 733 815 (76.0)	0.36 (0.35–0.37)
Simple contacts	7 099 (19.8)	1 458 201 (16.5)	1.25 (1.22–1.28)
Interactions	3 622 (10.1)	102 776 (1.2)	9.28 (8.97–9.60)
Referrals without consultation	4 185 (11.7)	368 380 (4.2)	3.02 (2.93–3.12)
Prescriptions	1 895 (5.3)	192 044 (2.2)	2.50 (2.39–2.62)
**Daytime contacts, age 6–11 years**	**43 082**	**7 498 501**	
Consultations	15 335 (35.6)	5 019 346 (66.9)	0.28 (0.27–0.28)
Simple contacts	9 574 (22.2)	1 508 783 (20.1)	1.13 (1.11–1.16)
Interactions	5 246 (12.2)	159 237 (2.1)	6.22 (6.04–6.40)
Referrals without consultation	6 546 (15.2)	364 849 (4.9)	3.46 (3.37–3.55)
Prescriptions	6 381 (14.8)	446 286 (6.0)	2.72 (2.65–2.80)
**Daytime contacts, age 12–17 years**	**29 476**	**7 154 661**	
Consultations	8 913 (30.2)	4 260 753 (59.6)	0.30 (0.29–0.30)
Simple contacts	6 753 (22.9)	1 795 233 (25.1)	0.89 (0.86–0.91)
Interactions	4 600 (15.6)	194 472 (2.7)	6.48 (6.29–6.69)
Referrals without consultation	2 562 (8.7)	223 391 (3.1)	2.93 (2.82–3.05)
Prescriptions	6 648 (22.6)	680 812 (9.5)	2.75 (2.68–2.83)

*Note*: Data are number (%) of daytime contacts for the three age groups. Bold values are total numbers for each age group.

GPs used ICPC-2 pain codes in 2 630 (6.1%) of the 43 302 consultations with cases, and in 1 902 399 (11.9%) of the 16 013 914 consultations with controls. GPs registered consultations with pain codes more frequently in older age groups both in cases (3.3 vs. 6.8 vs. 10.7%) and controls (5.1 vs. 15.3 vs. 18.6%) (Table 3). Similar findings were present in consultations with musculoskeletal pain codes (cases 1.7 vs. 3.5 vs. 6.3% and controls 1.8 vs. 7.4 vs. 11.7%). The risk that a consultation included a pain code was lower in cases than in controls at all ages (Table 4). The risk that a consultation included a pain code grouped as ‘musculoskeletal pain’ was lower in cases than in controls in the age groups 6–11 years and 12–17 years, while there was no difference between cases and controls in the age group 0–5 years. The risk for codes indicating headache and abdominal pain was lower in cases than in controls in all age groups. In cases, *Neurological disease, other* (N99), was the only RFE coded in 7 199 (16.6%) of 43 302 consultations.

**Table 3.** Daytime consultations given a pain related ICPC-2 code in children with CP (cases) and children in the general population (controls).

	Cases	Controls
**Daytime consultations, age 0–5 years**	**19 054**	**6 733 815**
All pain	631 (3.3)	341 855 (5.1)
Musculoskeletal pain	318 (1.7)	119 719 (1.8)
Other pain	313 (1.6)	222 136 (3.3)
**Daytime consultations, age 6–11 years**	**15 335**	**5 019 346**
All pain	1 045 (6.8)	768 234 (15.3)
Musculoskeletal pain	542 (3.5)	369 463 (7.4)
Other pain	503 (3.3)	398 771 (7.9)
**Daytime consultations, age 12–17 years**	**8913**	**4 260 753**
All pain	954 (10.7)	792 310 (18.6)
Musculoskeletal pain	563 (6.3)	499 452 (11.7)
Other pain	391 (4.4)	292 858 (6.9)

*Note:* Data are number (%) of all daytime consultations for all pain (musculoskeletal and other pain). Pain codes, as listed in Table 1, are included in ‘All pain’, ‘Musculoskeletal pain’ and ‘Other pain’. Bold values are total numbers of all consultations for each age group.

**Table 4.** Risk ratio analyses for all pain, headache, abdominal and musculoskeletal pain in children with CP (cases) compared to that in children in the general population (controls).

	Cases	Controls	Risk ratio (95%CI)
**All consultations, age 0–5 years**	**19 054**	**6 733 815**	
All pain	631	341 855	0.64 (0.59–0.69)
Headache	16	12 192	0.46 (0.28–0.76)
Abdominal pain	145	128 711	0.39 (0.33–0.46)
Musculoskeletal pain	318	119 719	0.94 (0.84–1.05)
**All consultations, age 6–11 years**	**15 335**	**5 019 346**	
All pain	1 045	768 234	0.41 (0.38–0.43)
Headache	79	75 916	0.34 (0.27–0.42)
Abdominal pain	248	254 521	0.31 (0.27–0.35)
Musculoskeletal pain	542	369 463	0.46 (0.42–0.50)
**All consultations, age 12–17 years**	**8 913**	**4 260 753**	
All pain	954	792 310	0.53 (0.49–0.56)
Headache	83	101 472	0.39 (0.31–0.48)
Abdominal pain	185	140 335	0.62 (0.54–0.72)
Musculoskeletal pain	563	499 452	0.51 (0.47–0.55)

*Note:* All pain includes all codes listed in Table 1. The following ICPC-2 codes were included in the three anatomical localizations: ‘Headache’ (N01 and N95), ‘Abdominal pain’ (D01, D02, D04 and D06) and ‘Musculoskeletal pain’ (as listed in Table 1). Bold values are total numbers of all consultations for each age group.

## Discussion

We found that children with CP accounted for 0.27% of the GPs’ paediatric daytime consultations, that GPs performed more administrative work for their paediatric patients with CP than for their other paediatric patients, and that in daytime consultations, pain was a less frequent RFE in children with CP than in the general paediatric population.

Our finding that cases accounted for 0.27% (or 2.7 per 1 000) of all daytime consultations corresponds with the national prevalence of CP (2.5 per 1000; 95% CI 2.4 − 2.7) [8]. In other words, as a group, the paediatric population with CP had a similar prevalence of GP consultations to that of the general paediatric population. In contrast, cases were overrepresented in reimbursement codes for GPs’ administrative work. This was as expected, since GPs have the authority to confirm their patients’ right to the majority of health and welfare benefits; in other words, they have a ‘door-keeper’ role in the Norwegian health care system. Still, in the age group 12–17 years controls had a higher risk for simple contacts than cases. An explanation may be that in 2016, new legislation required all high school students to provide a note from a GP if school absence was longer than two days. Populations with chronic health conditions, such as CP, were to some degree exempt from this rule.

We analysed only daytime contacts because we were interested in continuous care provided by the regular GP, as opposed to out-of-hour services. Our findings confirm that GPs are involved in the network of care for children with CP. Pain related codes were analysed in consultations only, because we were interested in contacts with the possibility of physical assessment in order to search for a cause of pain. The latter choice was supported by a Norwegian study that reported good correspondence between the patient record and diagnosis (ICPC-2 code) in consultations, but recommended caution if including simple contacts in the analysis of RFE in contacts with GPs [23].

The pain codes were less frequent in cases (6% of consultations) than in controls (12% of consultations). The ICPC-2 provides a choice to code an RFE as a symptom, a process, or a disease. In cases, the disease code *Neurological disorder (N99)* including CP was the only code in almost 17% of all daytime consultations. This finding might be a sign of inequity in coding between the population with CP and the general population. We hypothesize that in a busy clinical practice, an already established disease code could compete with a new ICPC-2 code for a current symptom or a process. Also, the reimbursement is not dependent on type or number of ICPC-2 codes. These factors might have caused an information bias. On the other hand, the complexity of chronic conditions may influence the caregivers’ expectations to a GP [25], and result in a preference to discuss recurrent pain during consultations in the specialist health care instead of during GP consultations. An indication for this is the finding in a previous study that Norwegian youth with CP contacted a GP only when their pain became severe [26].

The pain codes were more frequent in older age groups in both cases and controls. The latter finding is in accordance with studies on pain as an RFE in the general paediatric population [12], and on pain prevalence in the paediatric population with CP [10,14,27,28]. An explanation can also be that adolescents with chronic conditions such as CP go through a period of transition of health care, gradually ending regular follow-up in the specialist paediatric health care at the age of 18 years.

The risk for musculoskeletal pain as the RFE did not differ between cases and controls in the youngest age group, while older children with CP had a lower risk than controls. An explanation could be that the CP surveillance program in specialist health care includes assessment and treatment for musculoskeletal issues and movement disorders. Treatments such as botulinum toxin injections, intrathecal baclofen therapy, and corrective surgery in the limbs, are often offered in school age, and include follow-up in the specialist care. This could have reduced the need for a GP consultation for musculoskeletal pain in the older age groups.

The risk for a consultation for headache and abdominal pain was lower in cases than in controls in all three age groups. We do not have any reason to believe that such pain is less frequent in the population with CP. Thus, we hypothesize that in young people with CP, headache and abdominal pain were either not reported to a GP, or not coded by the GP.

This study has some limitations. First, we did not have information on mortality rate, emigration or parental socio-economic status. Other confounding variables may exist. Second, the data was too large to allow for longitudinal comparison on frequencies of daytime contacts and consultations on individual level among controls. Third, ICPC-2 uses the wording ‘symptom/complaint’ and seldom ‘pain’ in chapter L Musculoskeletal. We assumed that pain is the most common complaint/symptom in this organ system and therefore the most relevant reason for encounter with a GP. Further, information on frequent consulters among cases might have influenced our findings. This is a topic for future studies. Another topic for future research is GPs’ attitudes and knowledge regarding follow-up of children with chronic conditions such as CP. Collaboration between the multidisciplinary specialist team and GPs should be addressed in future research.

There are also several strengths of the study. NorCP has high completeness (76%) and high correctness (100%) of CP diagnosis [8]. Since Norwegian primary health care and specialist health care are state-funded, the market for private health services for children is limited. These factors ensure the generalizability of the study.

In conclusion, the study findings indicate that the potential of GPs’ involvement in pain management in paediatric CP is not fully exploited despite high availability and low costs. In order to improve pain management in CP, we advocate that all involved in the process of care take a proactive approach. In consultations, GPs should ask for pain even if the child with CP or parent does not address pain. Health care professionals in the local multidisciplinary team should encourage the family to consider consulting a GP if the child is in pain. Health care specialists should encourage the family to connect with a GP and a relevant patient organization. Simple measures such as introducing a symptom diary [29,30] whenever pain is recognized and offering feasible educational material might contribute to enhanced family empowerment, common language on pain and shared decisions on pain management.
